# Ancient Origin of Two 5S rDNA Families Dominating in the Genus *Rosa* and Their Behavior in the Canina-Type Meiosis

**DOI:** 10.3389/fpls.2021.643548

**Published:** 2021-03-08

**Authors:** Radka Vozárová, Veit Herklotz, Aleš Kovařík, Yuri O. Tynkevich, Roman A. Volkov, Christiane M. Ritz, Jana Lunerová

**Affiliations:** ^1^Department of Molecular Epigenetics, Institute of Biophysics, Academy of Sciences of the Czech Republic, Brno, Czechia; ^2^Department of Experimental Biology, Faculty of Science, Masaryk University, Brno, Czechia; ^3^Department of Botany, Senckenberg Museum of Natural History Görlitz, Görlitz, Germany; ^4^Department of Molecular Genetics and Biotechnology, Yuriy Fedkovych Chernivtsi National University, Chernivtsi, Ukraine; ^5^Chair of Biodiversity of Higher Plants, International Institute (IHI) Zittau, Technische Universität Dresden, Zittau, Germany

**Keywords:** 5S rDNA, evolution, *Rosa*, genomics, cytogenetics, repeatome, Rosaceae

## Abstract

The genus *Rosa* comprises more than 100 woody species characterized by intensive hybridization, introgression, and an overall complex evolutionary history. Besides many diploid species (2n = 2x = 14) polyploids ranging from 3x to 10x are frequently found. Here we analyzed 5S ribosomal DNA in 19 species covering two subgenera and the major sections within subg. *Rosa*. In addition to diploids and polyploids with regular meiosis, we focused on 5x dogroses (*Rosa* sect. *Caninae*), which exhibit an asymmetric meiosis differentiating between bivalent- and univalent-forming chromosomes. Using genomic resources, we reconstructed 5S rDNA units to reveal their phylogenetic relationships. Additionally, we designed locus-specific probes derived from intergenic spacers (IGSs) and determined the position and number of 5S rDNA families on chromosomes. Two major 5S rDNA families (termed 5S_A and 5S_B, respectively) were found at variable ratios in both diploid and polyploid species including members of the early diverging subgenera, *Rosa persica* and *Rosa minutifolia*. Within subg. *Rosa* species of sect. *Rosa* amplified the 5S_A variant only, while taxa of other sections contained both variants at variable ratios. The 5S_B family was often co-localized with 35S rDNA at the nucleolar organizer regions (NOR) chromosomes, whereas the co-localization of the 5S_A family with NOR was only exceptionally observed. The allo-pentaploid dogroses showed a distinct distribution of 5S rDNA families between bivalent- and univalent-forming chromosomes. In conclusion, two divergent 5S rDNA families dominate rose genomes. Both gene families apparently arose in the early history of the genus, already 30 myrs ago, and apparently survived numerous speciation events thereafter. These observations are consistent with a relatively slow genome turnover in the *Rosa* genus.

## Introduction

Ribosomal RNA genes (rDNA) encoding 5S, 5.8S, 18S, and 26S ribosomal RNA are ubiquitous in plants and are organized into arrays containing hundreds to thousands of tandem units at one or more genomic loci (Hemleben et al., 1988; Nieto Feliner and Rossello, 2012; Roa and Guerra, 2012). Each unit consists of an evolutionary conserved coding region of 120 bp and a variable intergenic spacer (IGS) (Long and Dawid, 1980). The units within the 5S arrays retain a high degree of identity due to homogenizing forces referred to as concerted evolution (Dover, 1982; Eickbush and Eickbush, 2007) where unequal crossing-over and gene conversion are major forces driving the process. Regardless of the mechanism, numerous factors such as the number of arrays, their mutation rate, formation of new variants, the intensity of natural selection, or efficient population size can affect homogenization of repeats (Dover, 1982; Ohta, 1984; Nagylaki, 1990). In plant hybrids and allopolyploids, homogenization of 5S rDNA arrays may not always occur as efficiently as that of 35S rDNA (Pedrosa-Harand et al., 2006; Weiss-Schneeweiss et al., 2008; Garcia et al., 2016; Amosova et al., 2019). As a consequence, two or more variants differing in the length and nucleotide sequence may simultaneously exist per genome (Cronn et al., 1996; Volkov et al., 2001, 2017; Fulnecek et al., 2002; Pastova et al., 2019; Benson et al., 2020).

The genus *Rosa* L. (Rosaceae) comprises about 150 species widely distributed across the northern hemisphere. Taxonomy is considered to be challenging because frequent polyploidy in app. 50% of the species (Yokoya et al., 2000; Roberts et al., 2009) and recurrent hybridization events may blur species boundaries (Ritz et al., 2005; Joly and Bruneau, 2006; Koopman et al., 2008). The existence of multiple cytotypes and variable degree of retention of progenitor alleles leading to incomplete lineage sorting complicating taxonomic classifications. In addition, species identification is generally hampered because most species are characterized rather by combinations of morphological characters than by single discriminating traits (Christ, 1873; Wissemann, 2003). Moreover, roses are one of oldest ornamentals (Wang, 2007) and their complex history of cultivation and breeding may generate another uncertainty in phylogenetic studies. Several attempts have been made to reconstruct the phylogeny of the genus (Millan et al., 1996; Matsumoto et al., 1998; Wissemann and Ritz, 2005; Bruneau et al., 2007; Zhang et al., 2013; Fougere-Danezan et al., 2015; Liu et al., 2015). Currently, the system comprises four subgenera: *Hulthemia* (one species), *Hesperhodos* (two species), *Platyrhodon* (one species), and *Rosa*, the latter consisting of 10 sections and comprising the vast majority of species (Wissemann, 2003). The most recent phylogenies detected *Rosa persica* (subg. *Hulthemia*) and *Rosa minutifolia* (subg. *Hesperhodos*) as early diverging lineages, and a major split of the genus into two large clades: the *Synstylae* and allies clade consisting of sect. *Synstylae*, *Indicae, Caninae, Bracteatae, Laevigatae*, and *Gallicanae* and the *Rosa* and allies clade comprising sect. *Rosa* [=*Cinnamomeae*] and *Pimpinellifoliae* (Wissemann and Ritz, 2005; Bruneau et al., 2007; Fougere-Danezan et al., 2015; Zhu et al., 2015; Debray et al., 2019).

The exclusively polyploid members of a large section *Caninae* (DC.) Ser. (dogroses), originated by multiple hybridization events (Ritz et al., 2005; Herklotz et al., 2018) represent a remarkable evolutionary lineage because they exhibit a peculiar unbalanced mode of sexual reproduction also known as Canina meiosis (Täckholm, 1920; Blackburn and Harrison, 1921). Canina meiosis results in a strongly matroclinal inheritance of genetic information since two pairing genomes form bivalents, while the remaining genomes remain unpaired as univalents and are transmitted by the female germ line only. Thus, at least hemisexual reproduction is ensured in the mostly pentaploid (2n = 5x = 35) species but tetraploids, hexaploids, and heptaploids also occur and their meiosis just differs by the number univalents (Wissemann, 2003; Roberts et al., 2009; Pachl, 2011). Amazingly, in plastid phylogenies, sect. *Caninae* appeared to be polyphyletic since species with fragrant glands (subsect. *Rubigineae* and *Vestitae*) were separated from the remaining species (subsect. *Caninae*) by *Rosa gallica* and *Rosa arvensis* which perform regular meiosis (Wissemann and Ritz, 2005; Fougere-Danezan et al., 2015). Thus, Canina meiosis has been probably evolved twice, which is supported by fluorescence *in situ* hybridization (FISH) analyses of meiotic chromosomes (Herklotz et al., 2018; Lunerova et al., 2020).

Ribosomal DNA loci have been studied in several diploid and polyploid species of *Rosa* so far. Ma et al. (1997) found one 35S rDNA locus per genome, located terminally on the short arms of small submetacentric chromosomes in five diploid species and one tetraploid cultivar of *Rosa*. Fernandez-Romero et al. (2001) found one 35S rDNA locus per genome at terminal locations on submetacentric chromosomes in five diploid species. These studies indicate the presence of a single nucleolar organizer regions (NOR) chromosome per haploid set of x = 7. In pentaploid dogroses, four to five 35S loci were reported implying the occasional loss of one locus (Lim et al., 2005; Herklotz et al., 2018). The 5S locus has been less commonly studied, while there is evidence for more than one 5S locus per haploid set. Two loci were found in the diploid *Rosa lucieae* [=*Rosa wichurana*] (Kirov et al., 2016), and some pentaploid dogroses may contain more than five sites (Lim et al., 2005; Herklotz et al., 2018) indicating a variable number of 5S loci per haploid set. The analysis of 5S rDNA clones from diploid *Rosa rugosa* revealed a conserved bipartite polymerase III promoter and non-coding IGS region (Tynkevich and Volkov, 2014b) evidencing that organization at the unit level is similar to most other plants. Analysis of 5S rDNA clones from four distantly related diploid species of *Rosa* (*R. nitida*, *R. rugosa*, *R. sericea*, and *R. lucieae*) showed a high level of intragenomic homogeneity. In contrast, the level of IGS similarity between *R. lucieae* and three other diploid species appeared to be unusually low (less than 58%) arguing for interspecies diversity in *Rosa* (Tynkevich and Volkov, 2014a, b).

In this study, based on genomic and cytogenetic approaches, we aim to map the evolutionary history of 5S rDNA loci across the genus *Rosa.* Based on available phylogenies of the genus, we selected 11 diploid and eight polyploid species representing the genus’ diversity (Table 1). Bioinformatic methods were used to determine the abundance and homogeneity of 5S rDNA in the genomes. Using locus-specific probes derived from 5S IGSs, we identified the two major 5S rDNA loci on the chromosomes by FISH.

**TABLE 1:** List of Rosaceae species used in this study, ploidy, source, and read archive accessions and the analyses employed.

Taxonomic rank^a^	Species/accession ID^b^	Ploidy	Methods applied^c^	Sequence read archive^d^/clone
Subgenus *Hesperhodos* COCKERELL	*R. minutifolia* ENGELM.	2x	Q, P, R	SRR7077023
Subgenus *Hulthemia* (DUMORT.) FOCKE	*R. persica* JUSS. [=*R. berberifolia* PALL.]	2x	Q, F, P, R	SRR7077021
Subgenus *Rosa*				
Sect. *Caninae* (DC). SER. subsect. *Caninae*	*R. canina* L. (CZ)	5x	Q, F, P, R	SRR8265808
	*R. canina* L. (DE-S27b)	5x	Q, P, R	ERR1662939
	*R. corymbifera* BORKH. (DE_2)	5x	Q, F, P, R	SRR8265810
	*R. dumalis* BECHST. (DE_34)	5x	Q, P, R	ERR1662941
Subsect. *Rubigineae* CHRIST	*R. inodora* FR. (DE_12)	5x	Q, F, P, R	ERR1662940
	*R. rubiginosa* L.	5x	Q, F, P, R	SRR10402274
Subsect. *Vestitae* CHRIST	*R. sherardii* DAVIES	5x	Q, P, R	SRR10402273
Sect. *Gallicanae* DC.	*R. gallica* L.	4x	Q, P, R	SRR6175524
Sect. *Indicae* THORY	*R. chinensis* JACQ.	2x	Q, P, R	SRR7077020
Sect. *Laevigatae* THORY	*R. laevigata* MICHX.	2x	Q, P, R	SRR7077018
Sect. *Synstylae* DC.	*R. arvensis* HUDS. (DE_8)	2x	Q, F, P, R	SRR8265809
	*R. lucieae* CRÉP. [=*R. wichurana* Crép.]	2x	Q, P, R	SRR6175519
	*R. multiflora* THUNB.	2x	Q, F, P, R	DRR059736
Sect. *Pimpinellifoliae* DC.	*R. spinosissima* L.	4x	Q, F, P, R	SRR8422951
Sect. *Rosa* [=*Cinnamomeae* DC.]	*R. majalis* HERRM. (DE_4)	2x	Q, F, P, R	SRR6175513
	*R. nitida* WILLD.	2x	F	n. d.
	*R. pendulina* L.	2x	Q, P, R	SRR6175522
	*R. rugosa* THUNB.	2x	Q, F, P, R	SRR6175514
Outgroups				
*Cliffortia curvifolia* WEIM.		2x	P	EU931716
*Acaena latebrosa* (AITON) W.T. AITON		2x	P	EU931698
*Geum urbanum* L.		2x	P	ERR2187925

*^*a*^Taxonomy within *Rosa* is according to Wissemann (2003). Species names are according to Plants of the world online (http://www.plantsoftheworldonline.org/); synonyms used in previous phylogenies of the genus are given in brackets. ^*b*^CZ, Czech Republic; DE, Germany. ^*c*^Q—5S families quantification by reads mapping, P—phylogeny tree construction, R—cluster analysis by RepeatExplorer, and F—fluorescent *in situ* hybridization. ^*d*^Sequence read archives (ENA/NCBI) submitted as parts of original projects (Herklotz et al., 2018; Nakamura et al., 2018; Saint-Oyant et al., 2018; Lunerova et al., 2020).*

## Materials and Methods

### Plant Material

Material of polyploid dogroses was sampled in wild populations in Germany and the Czechia (Supplementary Table S1). Diploid species and tetraploid species with regular meiosis were obtained from various Botanical Gardens (Supplementary Table S1). In addition, we retrieved sequence information from published work stored in the ENA database for bioinformatics analyses (Supplementary Table S1).

### Isolation and Cloning of 5S rDNA Sequences From *Rosa canina*

Total genomic DNA of *Rosa canina* was extracted from fresh leaves applying the standard protocol (Rogers and Bendich, 1985). The 5S rDNA repeats were amplified using the primers pr5S-14 and pr5S-15 (Tynkevich and Volkov, 2014b) with the 5’-extensions containing restriction endonuclease *Not*I recognition site. The PCR products were treated by *Not*I, ligated into the *Eco*52I recognition site of the pLitmus 38 plasmid, and used for transformation of *Escherichia coli* XL_blue line by electroporation method. Selected recombinant clones were sequenced using a BigDye Terminator Cycle Sequencing Kit (Thermofisher Scientific, United States). Clones containing inserts of A and B variants of 5S rDNA were identified by sequence analysis. The sequences were submitted to GenBank under the accession numbers MW349696 and MW349697.

The inserts of cloned 5S rDNA sequences contained genic regions and IGS. In order to increase the specificity of probe hybridization, we amplified the IGS sequences using specific primers annealing to 5S_A and 5S_B variants. The oligonucleotide primers’ sequences for the 5S_A IGS were as follows: A_for: 5′-CCTCTTTTTTCTGTTTCGGT-3′; A_rev: 5′-ATAAACTCCATTCGCTCAG-3′. Primers for the 5S_B variants were: B_for: 5′-ACCCCTCTTTTTGCCTTT-3′; B_rev: 5′-GCTTCGTCTCACTCCTCT-3’. The 25 μl PCR reaction contained 0.1 ng of plasmid DNA as the template, 4 pmol of each primer, 2.4 nmol of each dNTP, and 0.4 units of Kapa *Taq* DNA polymerase I (Kapa Biosystems). Cycling conditions were as follows: initial denaturation step (94°C, 180 s); 35 cycles (94°C, 20 s; 57°C, 30 s; and 72°C, 30 s). The length of amplified products was 373 nt for the A variant and 394 nt for the B variant. Purified PCR products were labeled by fluorescent dyes (as below) and used in FISH.

### Identification of 5S rDNA Sequences in High-Throughput Reads

**FIGURE 1:**
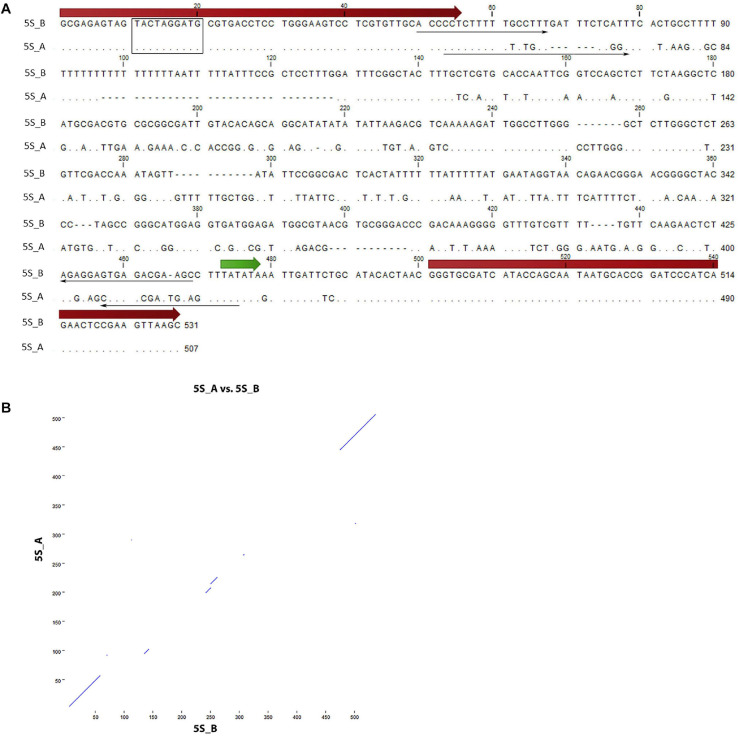
Sequence analysis of 5S rDNA clones from *Rosa canina*. **(A)** Alignment of the 5S_A and 5S_B clones. The position of 5S rRNA coding regions (thick arrows), primers (thin arrows), and regulatory regions (TATA box—in green, Box-C—rectangle) are highlighted. **(B)** Dot plot of pairwise comparisons clones. Note that only coding regions showed significant similarity.

For bioinformatic analyses, the whole genomic sequencing data for 19 *Rosa* accessions were used (Supplementary Table S2). The genome proportion of 5S rDNA families was determined using the total Illumina reads trimmed for quality (Phred score ≥ 30 over ≥ 95% read length). Trimmed reads (typically > 7 million) were mapped to corresponding 5S_A and 5S_B reference sequences (IGS subregion between the primers, Figure 1) using the following parameters: insertion and deletion costs_3, lengths fraction_0.5, similarity fraction_0.8, and deletion cost_2 (Qiagen, Germany). The distribution of SNPs across the 5S rDNA sequences was recorded when the distribution exceeded a threshold of at least 20 identical SNPs over at least 200 reads that covered the variant position and occurred at ≥ 10% frequency. For the alignment, minimal sequence length coverage was 50% and minimum sequence similarity was 90%. For the more distantly related species, *Rosa spinosissima* and *R. persica*, similarity threshold parameter was decreased to 80% (for the 5S_B variant). The genome abundance and copy number was calculated from genome proportions according to the formula stated in (Lunerova et al., 2020).

### Generation of Consensus Sequence and Phylogenetic Analyses

For phylogenetic reconstructions, the consensus 5S_A and 5S_B rDNA sequences were extracted from mapped reads using CLC genomic workbench. Additionally, we added the partial sequence of 5S ribosomal RNA genes from *Acaena latebrosa* (EU931698.1) and *Cliffortia curvifolia* (EU931716.1). Paired 250 bp Illumina reads of *Geum urbanum* (SRA accession ERR2187925) were mapped in a first round to the *C. curvifolia* (EU931716.1) sequence. Mapping was done with Bowtie2 (Langmead and Salzberg, 2012) implemented in Geneious^®^ 10.0.9^1^ with the lowest sensitivity pre-set. This resulted in two reads out of 22.8 million hitting to a 39 bp conserved region. The two reads were aligned and served in a second round with same parameters as mapping scaffold of 334 bp length. In the second mapping, 50 reads out of 22.8 million were assembled and its consensus was added to the alignment including all rose sequences and the two species from the *Acaena* clade. Alignments were done with MAFFT v7.450 algorithm (Katoh and Standley, 2013) implemented in Geneious using default parameters. Model test implemented in MEGA X v. 10.1.8 (Kumar et al., 2018) revealed the Tamura–parameter substitution model with invariant sites as most appropriate for the data based on Akaike information criterion (Tamura, 1992). Based on this model, we computed a maximum-likelihood tree in MEGA X whose branch support was evaluated by 1000 bootstrap replicates. Rooted with *G. urbanum*, this tree was used for the dating approach conducted with MEGA X. Therefore, we used two calibration points along the tree taken from the respective fossils given in Xiang et al. (2017) *Rosa germerensis*, 48.6 Mya (Edelman, 1975) and *Acaena* sp., 37.2 Mya (Zetter et al., 1999). A timetree inferred by applying the RelTime method (Tamura et al., 2018) was computed using two fixed calibration constraints. All positions containing gaps and missing data were eliminated (complete deletion option). A neighbor joining tree was constructed with the Seaview software (Gouy et al., 2010).

### Clustering Analysis of 5S rDNA

The fastq reads were initially filtered for quality and trimmed to uniform length using the pre-processing and QC tools in RepeatExplorer2 (Novak et al., 2013). Read length ranged between 100 and 150 bp, depending on sequencing library and the Illumina sequencing platform. After the fastq > fasta conversion and trimming to uniform length, reads were analyzed with the RepeatExplorer2 clustering program using default parameters. We used 1 million paired-end reads, or 1 million single-end reads as inputs for RepeatExplorer2 clustering. This bioinformatic pipeline runs a graph-based clustering algorithm (Novak et al., 2013) that assembles groups of frequently overlapping reads into clusters of reads, representing a repetitive element or part of a repetitive element with a higher order genome structure. The similarity and structure-based repeat identification tools in RepeatExplorer2 aid in identification of the repeats. RepeatExplorer2 uses a BLAST threshold of 90% similarity across 55% of the read to assign reads to clusters (minimum overlap = 55, cluster threshold = 0.01%, minimum overlap for assembly = 40), and the clusters are identified based on the principle of maximum modularity. We also used the SeqGrapheR (Novak et al., 2010) software in virtual space of Ubuntu 18.04 to visualize the specific reads corresponding to the 5S_A and 5S_B variants.

### Slide Preparation and FISH

For slide preparations, we used young anthers from flower buds of about 0.5 cm in length, which were harvested during spring 2019. Male meiosis was studied at prophase I (diplotene/diakinesis) where the bivalents and univalents could be easily distinguished from each other. Fresh flower buds were fixed using Carnoy solution (acetone:acetic acid, 2:1 or ethanol:acetic acid, 3:1 in some cases), and stored in 70% ethanol at −20°C. Before slide preparation, anthers were pre-treated by 0.5% PVP and 2% Triton-X100 (Sigma–Aldrich, United States) for 2–5 min followed by enzyme digestion overnight at 10°C in 1% cellulase, 0.2% pectolyase Y23, 0.5% hemi-cellulase, and 0.5%, macerozym R10 (Sigma–Aldrich, United States; Duchefa Biochemie, Netherlands) dissolved in citric buffer (0.04 M citric acid and 0.06 M sodium citrate). FISH followed the procedures described in Herklotz et al. (2018). Anthers were separated and squashed on slides in a drop of 70% acetic acid and fixed in liquid nitrogen.

For FISH, we used two probes derived from the 5S_A and 5S_B clones of the IGS region, respectively, and in addition, an 18S rDNA probe that was a 1.7-kb fragment of the 18S rRNA gene of *Solanum lycopersicum* (GenBank # X51576.1). The 5S rDNA genic region originated from *Artemisia tridentata* S4 clone, GenBank # JX101915.1. The probes were labeled by nick translation using Spectrum green dUTPs (Abbott, United States) for 5S_A rDNA, and Cy3-dUTPs (Roche, Switzerland) for 5S_B and 18S rDNA; 5S rDNA was labeled by Atto647N (Jena Bioscience, Germany). Slide preparation and hybridization followed standard protocols (Schwarzacher and Heslop-Harrison, 2000). Chromosomes were counterstained with 1 μg ml^–1^ DAPI (4’, 6-diamidine-2’-phenylindole dihydrochloride) diluted in mounting medium for fluorescence (Vectashield, Vector Laboratories, United Kingdom). The slides were scanned using epifluorescence microscopes (Olympus Provis AX70, with cold cube camera, Metasystems, Germany). Imaging software was ISIS (MetaSystems, Germany), and images were optimized for contrast and brightness with Adobe Photoshop CS6 and PS2020.

## Results

### Cloning and Sequencing of 5S rDNA Variants

Two 5S rDNA clones (5S_A and 5S_B) were isolated from *R. canina* IGS. Sequence analysis revealed some conserved regulatory elements: Box-C within the coding region, the TATA box at –29 (both clones), and T-rich terminators downstream of the coding sequences (Figure 1A). Box-A could not be unambiguously determined due to primer overlap. The 5S_B clone had a long (20 nt) T-tract which appears to be missing or was much shorter in clone 5S_A. By analogy with other 5S rRNA transcripts, the putative transcription started at the first G within the GGG motif following the C at −1 (Tynkevich and Volkov, 2014b). Intragenomic homogeneity was high, and no significant SNPs were revealed in mapping experiments (not shown). Pairwise alignment revealed conserved coding regions, while most of the IGS was dissimilar between both sequences (Figure 1B). We took advantage of considerable sequence divergence between both clones and amplified the locus-specific IGS subregions from plasmids. The resulting 373 bp (5S_A family) and 394 bp (5S_B family) PCR products were subsequently used in FISH.

### Representation of Individual 5S rDNA Variants in *Rosa* Genomes

**FIGURE 2:**
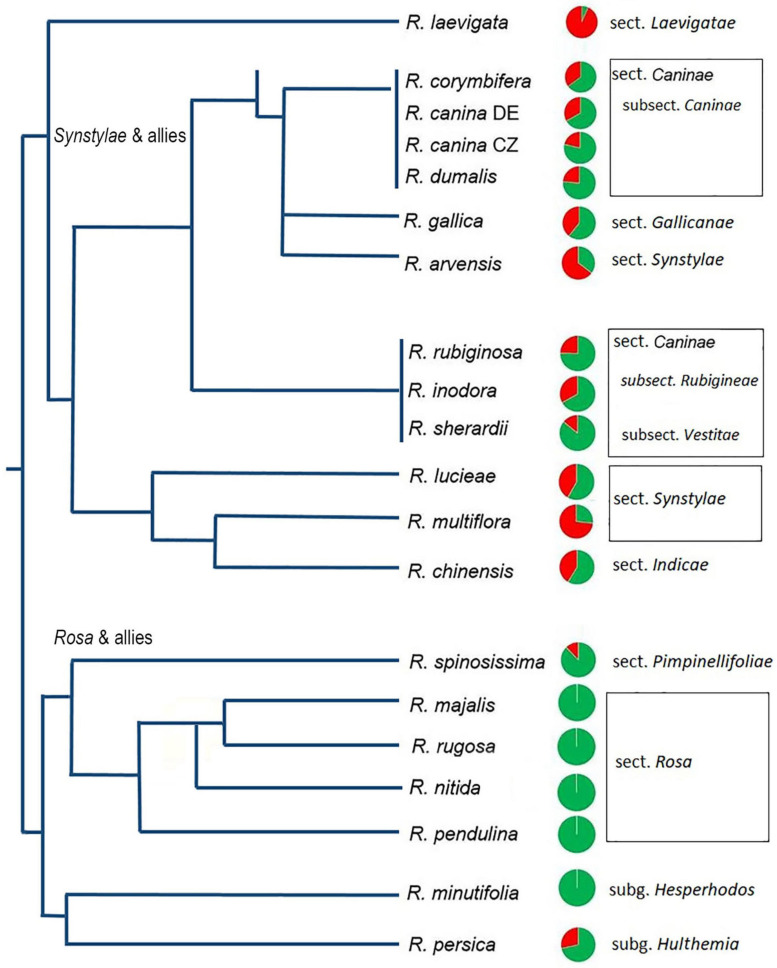
Quantitative relationships between 5S_A and 5S_B rDNA families in *Rosa* genomes. Genomic proportions of families calculated from high-throughput reads are shown as pie charts next to the species names. Green—5S_A family, red—5S_B family. Data are in Supplementary Table S3; data for *R. nitida* are taken from FISH (Supplementary Figure S5). A simplified phylogenetic tree is redrawn according to published phylogenies based on plastid sequences (Fougere-Danezan et al., 2015).

To determine the abundance of individual 5S rRNA gene families in *Rosa* genomes, we used available genomic resources (Table 1). The genome proportion of the 5S_A family was in average twice of that of the 5S_B family (Supplementary Tables S2, S3). The copy number per somatic cell (2C) ranged from 80–8000 (5S_A family) and 0–2400 (5S_B family). Both families appeared to be equally homogeneous containing a relatively low number of SNPs consistent with our previous findings obtained by comparison of individual 5S rDNA clones (Tynkevich and Volkov, 2014a, b). The contribution of each family to total 5S rDNA was expressed for each species by pie charts and visualized in a phylogenetic context (Figure 2). The diploid species from sect. *Synstylae*, all polyploid species, and *R. persica* (subg. *Hulthemia*) carried both families. *Rosa laevigata* contained the 5S_A family in low copy (c. 80 copies/2C), while its 5S rDNA was dominated by the 5S_B family (980 copies/2C) (Supplementary Table S3). Species from sect. *Rosa* and *R. minutifolia* (subg. *Hesperhodos*) carried the 5S_A rDNA family only. Blast searches failed to reveal significant hits of 5S_A and 5S_B sequences in genomic reads from *Prunus*, *Rubus*, *Fragaria*, *Cliffortia*, *Acaena*, and *Sanguisorba* (all Rosaceae) even at relaxed (e = 0.1) stringencies (not shown).

### Repeat Explorer Analysis of 5S rDNA Families in *Rosa* Genomes

**FIGURE 3:**
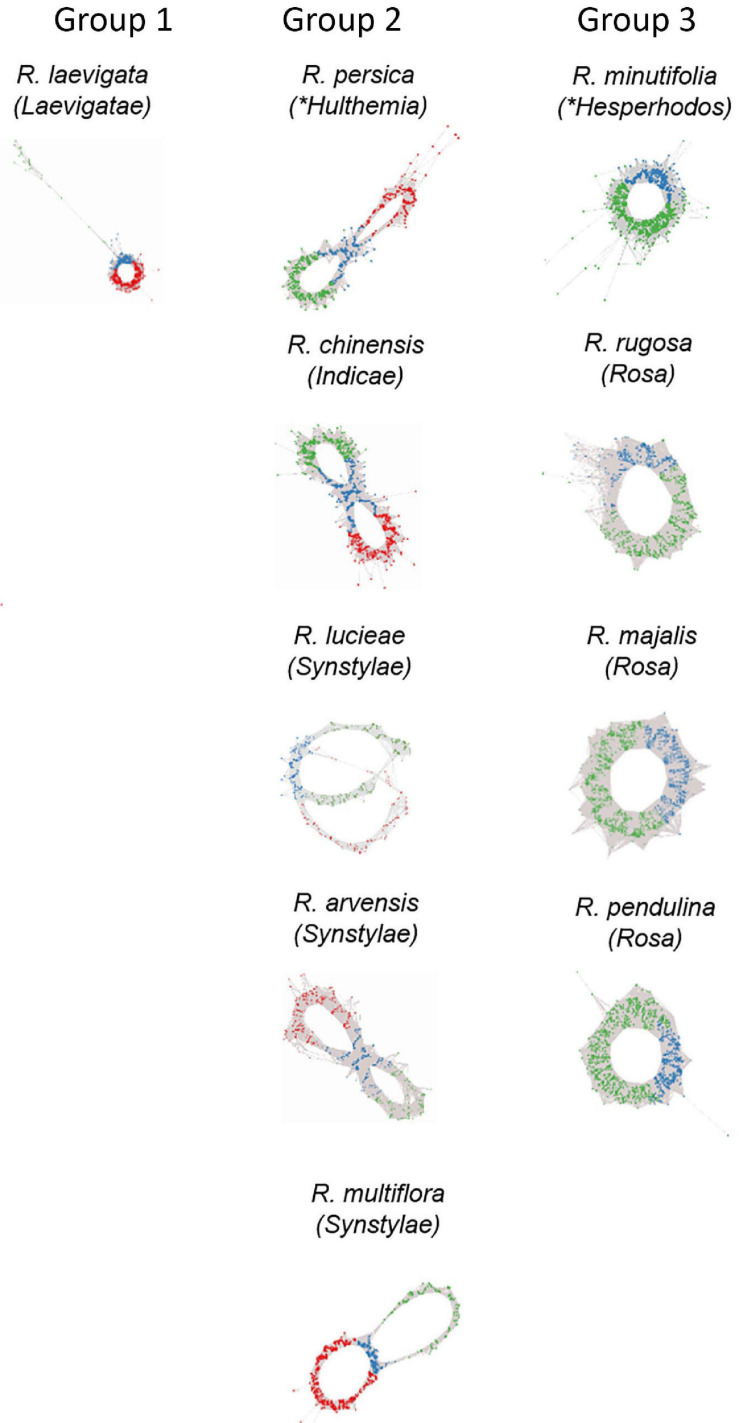
Genomic analysis of 5S rDNA variants in diploid (2n = 2x = 14) species of *Rosa*. Projections of 5S rDNA cluster graphs: Loops represent IGS reads colored according to the 5S_A (green) and 5S_B (red) IGS variants; genic regions are in blue. Group 1 represents species with dominance of 5S_B variant; Group 2 contains diploids with balanced ratio of both variants; and Group 3 shows species with only A variant in the genome. Name of the sections within subg. *Rosa* is given in brackets; other subgenera are marked by asterisks.

Cloning experiments cannot address the question about the distribution of gene families in the genome. Thus, in order to determine the number and genomic representation of individual 5S rRNA gene families, we applied clustering analysis (Figure 3). The cluster graph shapes provide information about the number and type of 5S gene families revealing potential hybridization and introgression (Garcia et al., 2020). It visualizes divergent IGS families as loops emanating from the bridge region which contains reads derived from a conserved coding region. Any loop can be considered as a separate gene family. The subregions in the graph can be annotated based on the read alignment against the 5S_A and 5S_B reference clones. Visual exploration indicates that there is no other 5S family amplified except of 5S_A and 5S_B types. The cluster graphs obtained from different *Rosa* genomes were categorized based on their structure into three groups (Figure 3). Group 1 comprised a single species *R. laevigata* (sect. *Laevigatae*) with predominant 5S_B type family representation. Group 2 comprised *R. persica* (subg. *Hulthemia*) and the majority of diploid species (sect. *Synstylae* and *Indicae*) showing a typical two-loop structure representing relatively balanced ratios of both families. Group 3 contained the diploid species *R. minutifolia* (subg. *Hesperhodos*) and species of sect. *Rosa* harboring a single 5S rDNA family (A). All polyploid species (both, those with regular meiosis and those with Canina meiosis) showed a Group 2 profile indicating the presence of both A and B families (Supplementary Figure S1). In sum, quantitative relationships between both 5S rDNA families were confirmed. Moreover, cluster analysis showed that the maximum number of 5S rDNA families in the *Rosa* genomes is always two, irrespective of the ploidy level.

### Phylogenetic Relationships Between 5S rDNA Families

**FIGURE 4:**
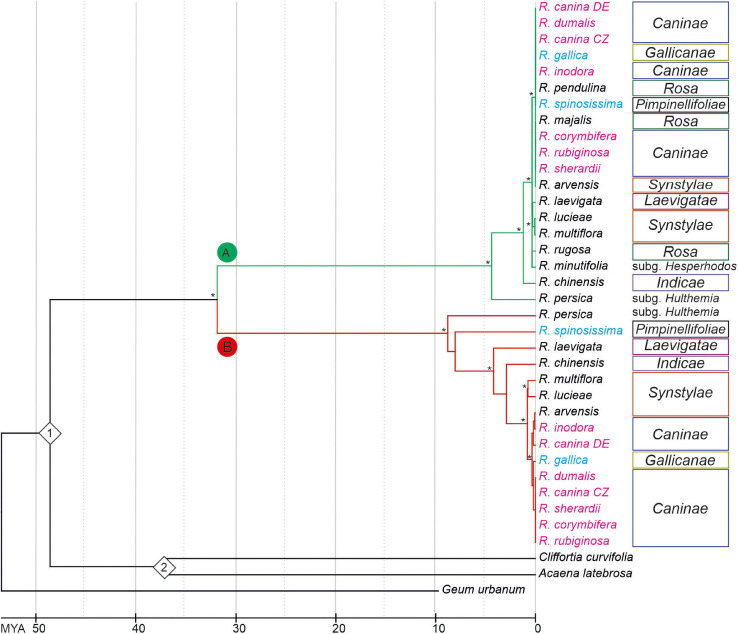
Chronogram based on a phylogeny among *Rosa* species inferred from Maximum-Likelihood analysis of 5S rDNA intergenic spacers. The tree was rooted with *Geum urbanum*. Bootstrap value of ≥ 70% is indicated by an asterisk. Species names are colored according to ploidy level (2x—black, 4x—light blue, 5x—pink). Color of branches represents 5S_A (green) and 5S_B (red) variants of IGS rDNA. Calibration points (1 = *Rosa gemerensis*; 48.6 myrs and 2 = *Acaena* sp.; 37.2 myrs) are indicated by diamonds.

To determine the phylogenetic relationships between 5S rDNA families, we computed phylogenies based on aligned 5S rDNA consensus sequences (obtained from mapping experiments. Both the maximum-likelihood (Figure 4 and Supplementary Figure S2) and neighbor joining (Supplementary Figure S3) trees separate the A and B 5S rDNA families clearly into two well-supported clades (A and B). Both, clades A and B contained diploid and polyploid species. Except of sect. *Rosa*, whose members clustered exclusively within the A clade, members of other sections, including *Synstylae*, *Indicae*, *Laevigatae*, *Pimpinellifoliae*, and *Caninae*, partitioned their 5S rDNA between both clades. Sequences from *R. persica* (subg. *Hulthemia*) were consistently positioned on early diverging nodes at both subclades. The major 5S rDNA family of *R. laevigata* (sect. *Laevigatae*) branched off at a rather basal position in clade B. The 5S_B family of 4x *R. spinosissima* positioned as sister to *R. persica*. Five 5S rDNAs of 5x species from sect. *Caninae* clustered together in both clades with negligible resolution between species (Supplementary Figures S2, S4). Out of the diploids, 5S sequences of *R. arvensis* (sect. *Synstylae*, B clade) and *R. pendulina* in (sect. *Rosa*, A clade) were most closely related to those of the respective *Caninae* branches. To gauge the length of time these 5S rDNA variants have existed in the *Rosa* genomes, we used two calibration points (48.6 myrs for *R. germerensis* and 37.2 myrs for *Acaena* sp.). We estimated that a common ancestor of both A and B families lived about 32 myrs ago (Figure 4) relatively long before separation of modern clades.

### FISH Analysis of 5S rDNA Variants on Chromosomes

**FIGURE 5:**
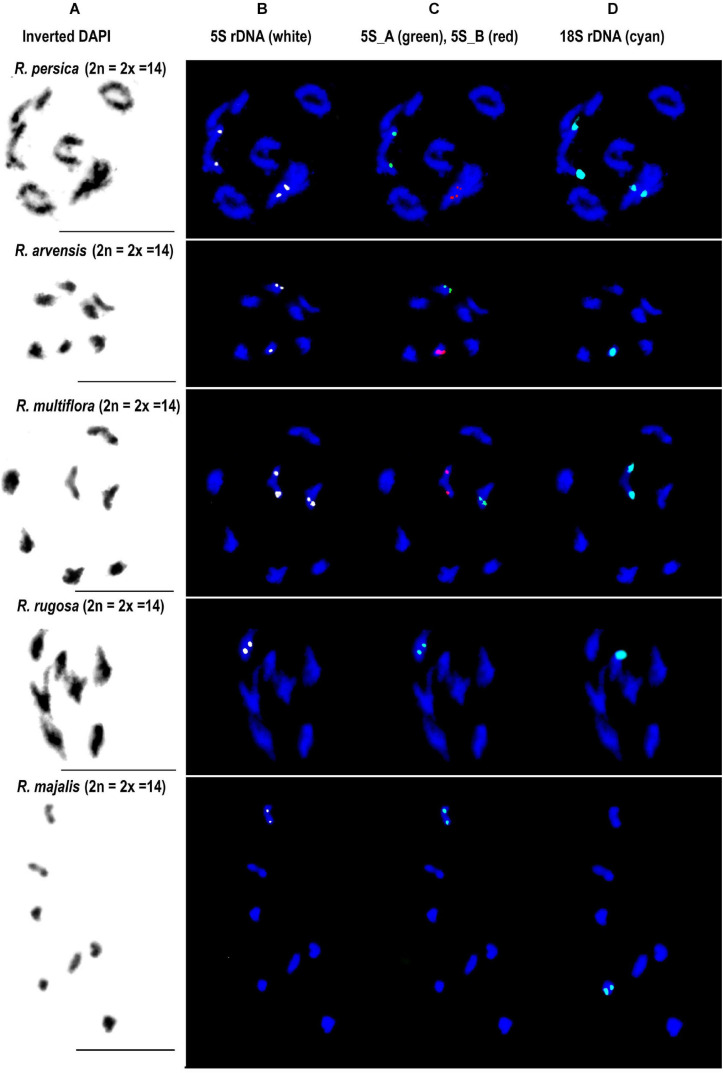
FISH analysis of 5S rDNA in diploid species. Diakinesis of meiotic phase I shown in *R. persica* (subgenera *Hulthemia*), *R. arvensis and R. multiflora* (section *Synstylae*), *R. rugosa* and *R. majalis* (section *Rosa*). In each species, the same metaphase was hybridized with the 5S genic **(B)**, 5S intergenic **(C)**, and 18S rDNA **(D)** probes. **(A)** DAPI staining (gray scale, inverted) of chromatin. Scale bar: 10 μm.

**FIGURE 6:**
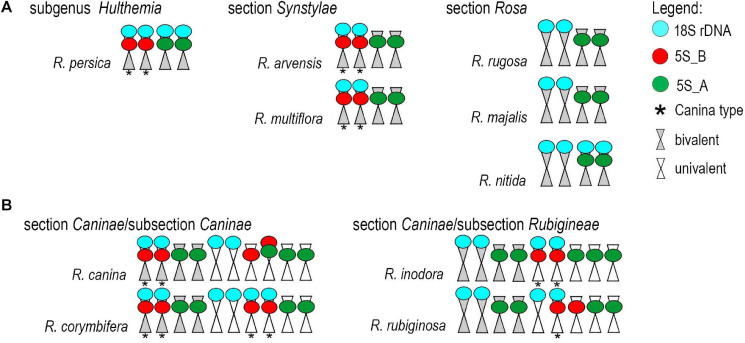
Ideograms of chromosomes carrying the rDNA loci. **(A)** Diploid species. **(B)** Pentaploid species from section *Caninae*.

Fluorescence *in situ* hybridization was conducted to visualize the position and number of 5S rDNA variants on chromosomes in several diploid and polyploid species. The diploids included representatives of subg. *Rosa* sect. *Synstylae* (*R. arvensis* and *R. multiflora*), sect. *Rosa* (*R. rugosa, R. majalis*, and *R. nitida*), and subg. *Hulthemia* (*R. persica*). Meiotic chromosomes from anthers (Figure 5A) were hybridized with rDNA probes derived from the 5S rDNA genic (Figure 5B, shown in white), 5S_B (red), and 5S_A (green) IGS subregions (Figure 5C). Additionally, the same chromosome spreads were re-hybridized with the 18S rDNA probe (shown in cyan, Figure 5D). In *R. arvensis* and *R. multiflora*, each 5S_A and 5S_B probe hybridized to one bivalent (one pair of chromosomes). The 5S_B probe was always co-localized on a chromosome bearing also the 18S rDNA signal. In *R. rugosa* and *R. majalis*, the 5S_A probe hybridized to a single bivalent, while we did not detect any hybridization signals with the 5S_B probe in accordance to genomic analyses (Figures 2, 3). In both species, the 18S and 5S rDNA loci were separate. However, in *R. nitida*, the 5S_A probe hybridized to one pair of chromosomes (mitotic metaphase from root tips, Supplementary Figure S5) which carried 18S rDNA signal (NOR). In *R. persica*, both variant-specific 5S rDNA probes hybridized to a single bivalent each, and these bivalents carried also 18S rDNA sites. The number and position of rDNA loci on chromosomes are summarized in Supplementary Table S4 and are diagrammatically depicted by ideograms (Figure 6).

**FIGURE 7:**
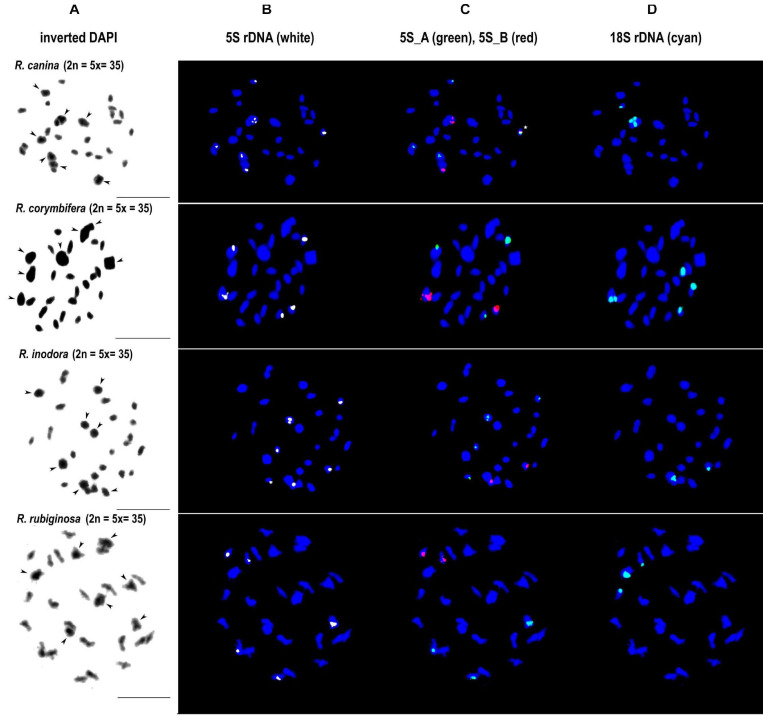
FISH analysis of meiotic chromosomes in pentaploid dogroses section *Caninae*: *R. canina* and *R. corymbifera* (subsection *Caninae*); *R. inodora* and *R. rubiginosa* (subsection *Rubigineae*). In each species, the same diakinesis was hybridized with the 5S genic **(B)**, 5S intergenic **(C)**, and 18S rDNA **(D)** probes. DAPI staining (gray scale, inverted) is to the left margin **(A)**. Arrowheads indicate bivalent chromosomes. In *R. canina*, a chromosome with co-localized 5S_A and 5S_B signals is marked with an asterisk. Scale bar: 10 μm.

We further analyzed meiotic (Figure 7) and mitotic (Supplementary Figure S5) chromosomes in polyploid species. As expected meiotic chromosomes of four 5x dogrose species (sect. *Caninae*) were represented by seven bivalents (pairs of chromosomes) and 21 univalents (Figure 7). In *R. canina* and *R. corymbifera* (subsect. *Caninae*), the 5S_A probe hybridized to one bivalent and three sites on univalent chromosomes. The 5S_B probe hybridized to one bivalent carrying the 18S (NOR) signal and two sites on univalents. The 5S_A and 5S_B signals occurred on different chromosomes except of one univalent chromosome in *R. canina* where both probes were co-localized. In *R. inodora* (subsect. *Rubigineae*), the 5S_A probe hybridized to one bivalent and three univalent chromosomes. The 5S_B probe hybridized to two univalent chromosomes carrying the 18S rDNA signal. *Rosa rubiginosa* (subsect. *Rubigineae*) showed a similar distribution of signals like *R. inodora* except that only one out of two 5S_B univalent chromosomes co-localized with the 18S signal. In addition, there were only two 5S_A sites on univalents. Collectively, these observations indicate that the number of rDNA sites, their chromosome position, and their meiotic behavior differ between subsections *Caninae* and *Rubigineae*. FISH on mitotic chromosomes from 4x *R. spinosissima* is shown in Supplementary Figure S5. In this species, the 5S_B probe hybridized to a chromosome pair which also carried the 18S rDNA signal (NOR) (Supplementary Figure S5). Two other 5S_B signals were colocalized (but did not overlap) with that of 5S_A on non-NOR chromosomes. 18S rDNA and 5S_A signals were localized on two separate chromosomes. Results are summarized in Figure 6 and Supplementary Table S4.

## Discussion

To study chromosome evolution and potential hybridization events in the genus *Rosa*, we analyzed the structure and organization of 5S rDNA in several diploid and polyploid species. We found that the genus is dominated by essentially two 5S rDNA families which markedly differ in IGSs and date back to the genus’ base.

### Ancient Origin of 5S rDNA Variants in the Genus *Rosa*

The IGSs of rRNA genes are rapidly evolving sequences, and it is common to find variation even between closely related species. It was therefore striking to observe that the genus *Rosa* is dominated essentially by only two 5S rDNA families and that no other family was amplified in any of the species analyzed here. Both families occupy different chromosome loci: the 5S_B family was always co-localized with NOR (35S rDNA), while the 5S_A family was mostly but not exclusively (see below) separate (Figure 6). Moreover, *R. persica* (subg. *Hulthemia*) amplified both families at similar ratio in its genome (Figures 2, 3). In contrast to all other diploid species (Ma et al., 1997), *R. persica* is also exceptional in possessing two NORs instead of one per haploid chromosome set. Since *R. persica* was consistently identified as the earliest divergent lineage in most phylogenies (Fougere-Danezan et al., 2015; Debray et al., 2019), we presume that the configuration with NORs co-localizing with distinct 5S rDNA families (Figure 6) is an ancient condition, while the NOR chromosome without 5S rDNA locus is derived. This assumption is supported by the following observations: First, the *Rosa* and allies clade contained the 5S_A family which was co-localized with 18S rDNA locus in *R. nitida* but not in *R. majalis* and *R. rugosa.* Second, all members of *Synstylae, Indicae*, and *Pimpinellifoliae* contained two 5S rDNA families albeit at differing ratios. For example, *R. multiflora* (sect. *Synstylae*) had prevalent 5S_B family, while the 5S_A family dominated in *R. chinensis*, a member of the closely related sect. *Indicae*. Similarly, in *R. lucieae* ([*R. wichurana*]; sect. *Synstylae*), both families are likely to be represented by two loci out of which one co-localized with 18S rDNA on the same chromosome (Kirov et al., 2016). Third, *R. laevigata* (sect. *Laevigatae*) was dominated by the 5S_B family (Figures 2, 3). This species is sister to the remaining species of the *Synstylae* and allies clade (Fougere-Danezan et al., 2015; Debray et al., 2019).

Of note, *R. nitida* differed from *R. majalis* and *R. rugosa* in having the 5S_A family co-localized with NOR. Traditionally, *R. nitida* has been classified into a separate section called *Carolinae* (Crépin, 1889). However, more recent taxonomies based on molecular markers failed to support this distinction and all three species are now placed within the *Rosa* and allies clade (Wissemann and Ritz, 2005; Joly and Bruneau, 2006). Interestingly, members of sect. *Rosa* tend to have much smaller loci of the centromeric satellite repeat CANR4 compared to other species of the genus (Lunerova et al., 2020). However, *R. nitida* is exceptional in having large abundance of the CANR4 satellite (10 out 14 chromosomes carried strong FISH signals, not shown). These features suggest chromosomal rearrangements accompanying speciation events in sect. *Rosa* although the basic chromosome number (x = 7) remained unchanged.

Neither A nor B type sequences were found in 5S rDNA of the genera *Prunus*, *Rubus*, *Fragaria*, *Cliffortia*, *Acaena*, and *Sanguisorba* (all Rosaceae). These observations suggest that both 5S rDNA families have their origin in the early evolution of the genus *Rosa* because the common ancestor of both families was dated at app. 32 myrs ago. Furthermore, molecular dating of diversification within both 5S rDNA clades was dated to app. 5–9 myrs each (Figure 4) matching at least roughly the origin of *Synstylae* and allies and *Rosa* and allies (Fougere-Danezan et al., 2015).

### The Fate of 5S rDNA in Allopolyploid Dogroses

Despite considerable interest, the composition of pentaploid (2n = 5x = 35) dogrose genome remains enigmatic (Wissemann, 1999; Nybom et al., 2004; Ritz et al., 2005; Herklotz et al., 2018; Lunerova et al., 2020). Previous studies based on microsatellite markers revealed genetic distinction between bivalent- and univalent-forming chromosomes (Nybom et al., 2006). The analysis of 35S rDNA markers confirmed these assumptions revealing two highly divergent ITS types (named Canina type and Rubiginosa type) present at variable ratios in subsections *Caninae* and *Rubigineae*, respectively (Ritz et al., 2005; Kovařik et al., 2008). Here we show that the *Canina* type ITS is co-localized with 5S_B rDNA locus (NOR), while the *Rubiginosa* ITS type is not. This Canina type of configuration [equivalent to marker chromosome 1 (Lim et al., 2005) or chromosome 7 (Kirov et al., 2016)] is typical for the bivalent-forming chromosomes in *R. canina* and *R. corymbifera*, both from subsection *Caninae* (Figure 6). In contrast, the Canina-type configuration of both rDNAs is found on univalent chromosomes in *R. rubiginosa* and *R. inodora* (subsection *Rubigineae*). The bivalent-forming chromosomes in this subsection carry the 5S_A family on a non-NOR chromosome. These data support the hypothesis that dogroses from both subsections arose by reciprocal hybridization of closely related species (Bruneau et al., 2007; Herklotz and Ritz, 2017). A very similar composition of dogrose genomes is also supported by the shallow nodes among dogroses in the 5S rDNA phylogeny (Figure 4). However, within the subsections, we detected some variation in number and distribution of loci especially between the univalent sets. For example, *R. inodora* carried two univalent-forming chromosomes with Canina type configuration, while *R. rubiginosa* contained only one. Additionally, one of the *R. canina* univalent chromosomes carried both 5S_A and 5S_B chromosomes co-localized, while this chromosome was not found in the closely related *R. corymbifera*. The variation between univalent chromosomes is consistent with increased diversity of microsatellite markers on univalent genomes (Nybom et al., 2004) and could be either related to divergence of genome donors and/or to partial degeneration of univalent chromosomes due to their exclusion from recombination in meiosis. Although 5S rDNA pseuodogenes seem to be present in *R. rugosa* (Tynkevich and Volkov, 2014b), there is no indication for extensive 5S rDNA pseuodogenization in *R. canina* (Lim et al., 2005) and in other dogroses (this work).

Although it might be preliminary to trace potential genome donors of allopolyploid dogroses, it is notable that the tetraploid species with a regular meiosis, *R. gallica* (sect. *Gallicanae*), tends to cluster with dogroses. It is therefore possible that pentaploid dogroses actually arose by pollination of an unreduced non-dogrose tetraploid egg cell by a reduced male gamete from a diploid donor. In support, *R. gallica* ITS types were occasionally found in dogroses (Herklotz et al., 2018) and *R. gallica* belonged to a clade together with dogroses in plastid phylogenies (Fougere-Danezan et al., 2015). Moreover, *R. gallica* shares the distinct morphological feature of partially pinnate sepals with dogroses, which is absent in the remaining species of the genus. However, there are also tetraploid cytotypes within dogroses (e.g., *R. villosa* L., *R. canina*) forming triploid egg cells and haploid sperm cells, which would not fit in the proposed scenario so far but might have arisen by another combination of partially reduced gametes. The also occurring higher ploidy (6x) levels which are less frequently found in dogroses rather originated from hybridizations within dogrose species involving unreduced egg cells (Herklotz and Ritz, 2017).

### Potential Factors Influencing Genetic Stability of 5S rDNA Loci in Roses

The maintenance of two 5S rDNA families in the *Rosa* genomes is consistent with the increased stability of 5S loci as compared to 35S loci in allopolyploid genomes documented in several allopolyploid systems (Pedrosa-Harand et al., 2006; Weiss-Schneeweiss et al., 2008; Garcia et al., 2016; Amosova et al., 2019). The reasons for relative stasis of 5S rDNA loci are not well understood, while their position on chromosomes (Garcia et al., 2016) and epigenetic modifications (5S rDNA loci carry mostly heterochromatic landmarks; Simon et al., 2018) have been discussed. One also has to consider the relative scarcity of meiosis driving genetic recombination (and homogenization) in these long-living perennial shrubs. For example, a *R. canina* individual known as “Rose of Hildesheim” (North Germany) is estimated to be more than 700 years old (Peters and Peters, 2013). Interestingly, *Gossypium* allopolyploids which represent also perennial shrubs tend to maintain 5S rDNA loci relatively intact over millions of years, while they homogenized their 35S rDNA loci (Cronn et al., 1996).

## Conclusion

We identified two 5S rDNA families which are widespread across the *Rosa* genus. The molecular and cytogenetic observations lead us to propose that both families have their origin deep in the genus history probably close to its base. A remarkably slow tempo of 5S rDNA evolution differs from other systems where these loci show considerable dynamics. The retention of a large number of ancient rDNA sequences in *Rosa* genomes resonates with drastic allelic heterozygosity encountered in previous studies of microsatellites (Nybom et al., 2006), protein coding genes sequences (Joly and Bruneau, 2006), and more recent whole genome sequencing projects (Raymond et al., 2018). In future, it will be interesting to analyze expression of alleles inherited from deep evolutionary times.

## Data Availability Statement

The datasets presented in this study can be found in online repositories. The names of the repositories and accession number(s) can be found below: https://www.ncbi.nlm.nih.gov/genbank/, MW349696; https://www.ncbi.nlm.nih.gov/genbank/, MW349697.

## Author Contributions

AK, CR, and RAV conceived and designed the study. CR, JL, RAV, VH, and YT performed the experiments and collected material. JL, RV, RAV, VH, and YT analyzed the data. AK, CR, JL, and RAV wrote the manuscript. All authors contributed to the article and approved the submitted version.

## Conflict of Interest

The authors declare that the research was conducted in the absence of any commercial or financial relationships that could be construed as a potential conflict of interest.

## Footnotes

1 https://www.geneious.com

## Supplementary Material

Supplementary Figure 1 Genomic analysis of 5S rDNA variants. Projections of 5S rDNA cluster graphs in polyploid *Rosa* species. Loops representing the IGS reads are colored according to the 5S_A (green) and 5S_B (red) IGS sequences. Blue nodes represent the 5S genic regions.

Supplementary Figure 2 Phylogenic relationships among *Rosa* species inferred from Maximum-Likelihood analyses of 5S rDNA intergenic spacers. The tree was rooted with *Geum urbanum*. Species names are colored according to ploidy level (2x—black, 4x—light blue, 5x—pink). Branch colors represent 5S_A (green) and 5S_B (red) lineages. Bootstrap support >70% is indicated above branches.

Supplementary Figure 3 A Neighbor-Joining phylogeny tree constructed from the 5S rDNA consensus sequences. The same data set as in Figure 4 was analyzed using the Juke Cantor model. One thousand repetitions were allowed for the statistical support. Bootstrap support levels >80% are indicated.

Supplementary Figure 4 Phylogenetic NJ trees constructed from type A and B 5S rDNA sequences, respectively. Note a similar position of *R. persica* on both trees (early separating). Note clustering of *Caninae* species (red asterisks after the names) in an unresolved branch. Note incongruent placement of the *R. spinosissima* variants.

Supplementary Figure 5 FISH analysis of root tip mitotic chromosomes from *R. spinosissima* and *R. nitida*. Arrows indicate NOR chromosomes with co-localized 5S rDNA loci. Note odd (5) number of 5S rDNA sites in the assumingly *R. spinosissima* allotetraploid.

Supplementary Table 1 Information about the taxonomy and origin of plants used in this study.

Supplementary Table 2 Details of bioinformatic procedures leading to 5S rDNA genome proportion and copy number estimation. Sheet 1—5S_A variant. Sheet 2—5S_B variant.

Supplementary Table 3 Summary of genome proportions and copy number of 5S rDNA families in *Rosa* genomes.

Supplementary Table 4 Summary of cytogenetic FISH analyses.
