# Supplementary figures and images for: Ancient Origin of Two 5S rDNA Families Dominating in the Genus Rosa and Their Behavior in the Canina-Type Meiosis
Source: Front Plant Sci. 2021 Mar 8;12:643548. doi: 10.3389/fpls.2021.643548 (PMC7984461; doi:10.3389/fpls.2021.643548)

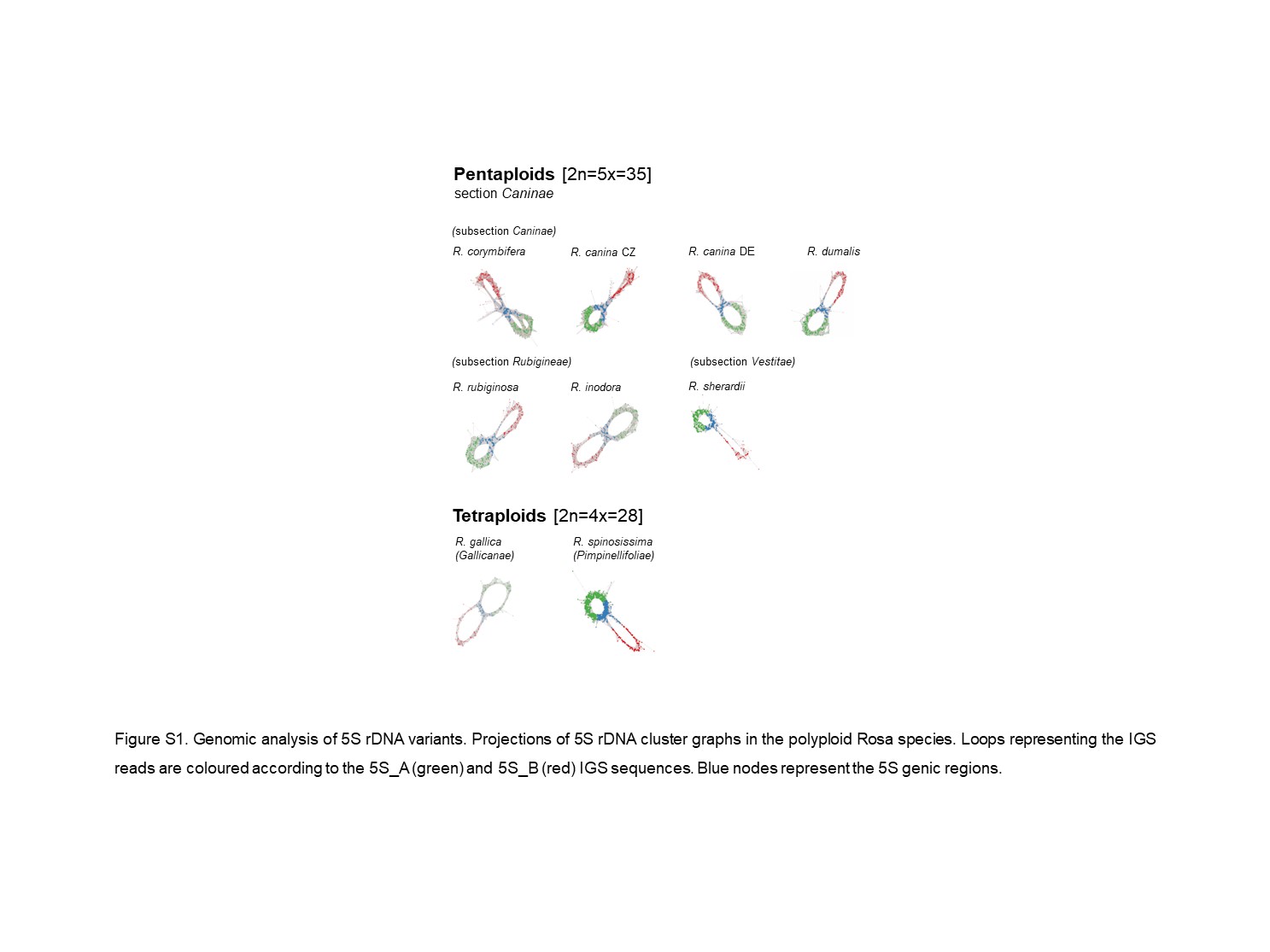

Supplement: Supplementary Figure 1 — Genomic analysis of 5S rDNA variants. Projections of 5S rDNA cluster graphs in polyploid Rosa species. Loops representing the IGS reads are colored according to the 5S_A (green) and 5S_B (red) IGS sequences. Blue nodes represent the 5S genic regions. [file Image_1.jpg]

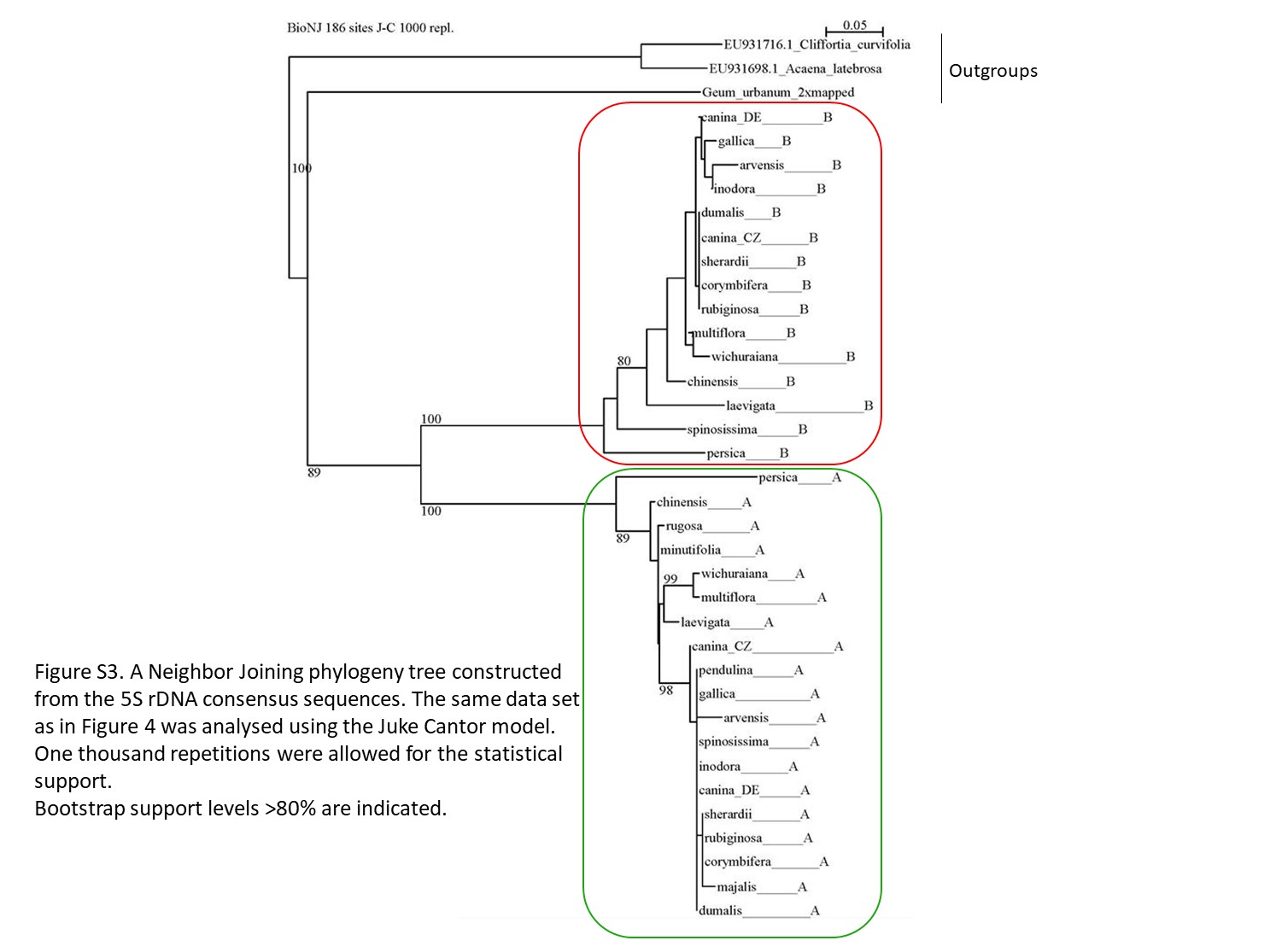

Supplement: Supplementary Figure 3 — A Neighbor-Joining phylogeny tree constructed from the 5S rDNA consensus sequences. The same data set as in Figure 4 was analyzed using the Juke Cantor model. One thousand repetitions were allowed for the statistical support. Bootstrap support levels >80% are indicated. [file Image_3.jpg]

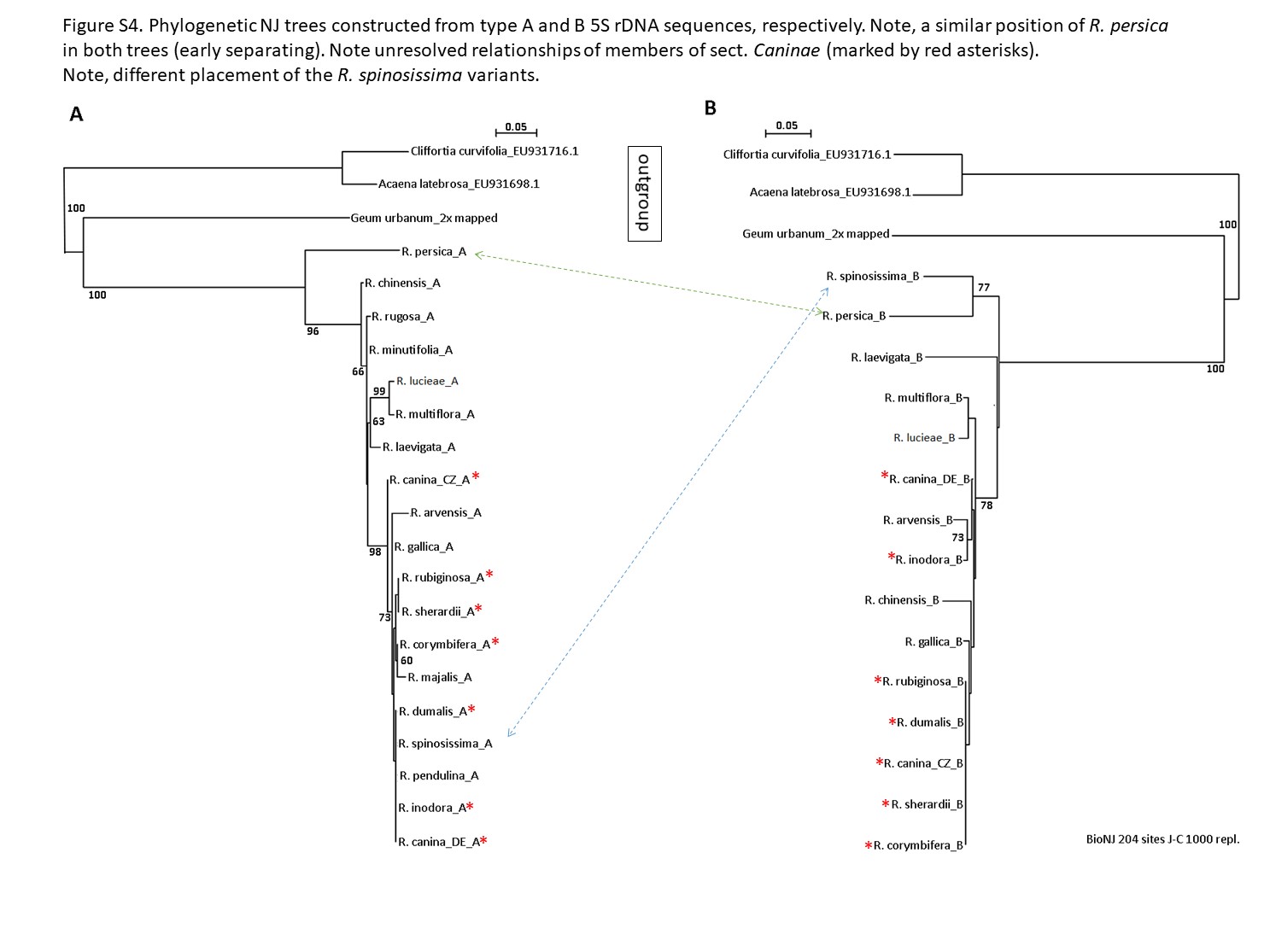

Supplement: Supplementary Figure 4 — Phylogenetic NJ trees constructed from type A and B 5S rDNA sequences, respectively. Note a similar position of R. persica on both trees (early separating). Note clustering of Caninae species (red asterisks after the names) in an unresolved branch. Note incongruent placement of the R. spinosissima variants. [file Image_4.jpg]

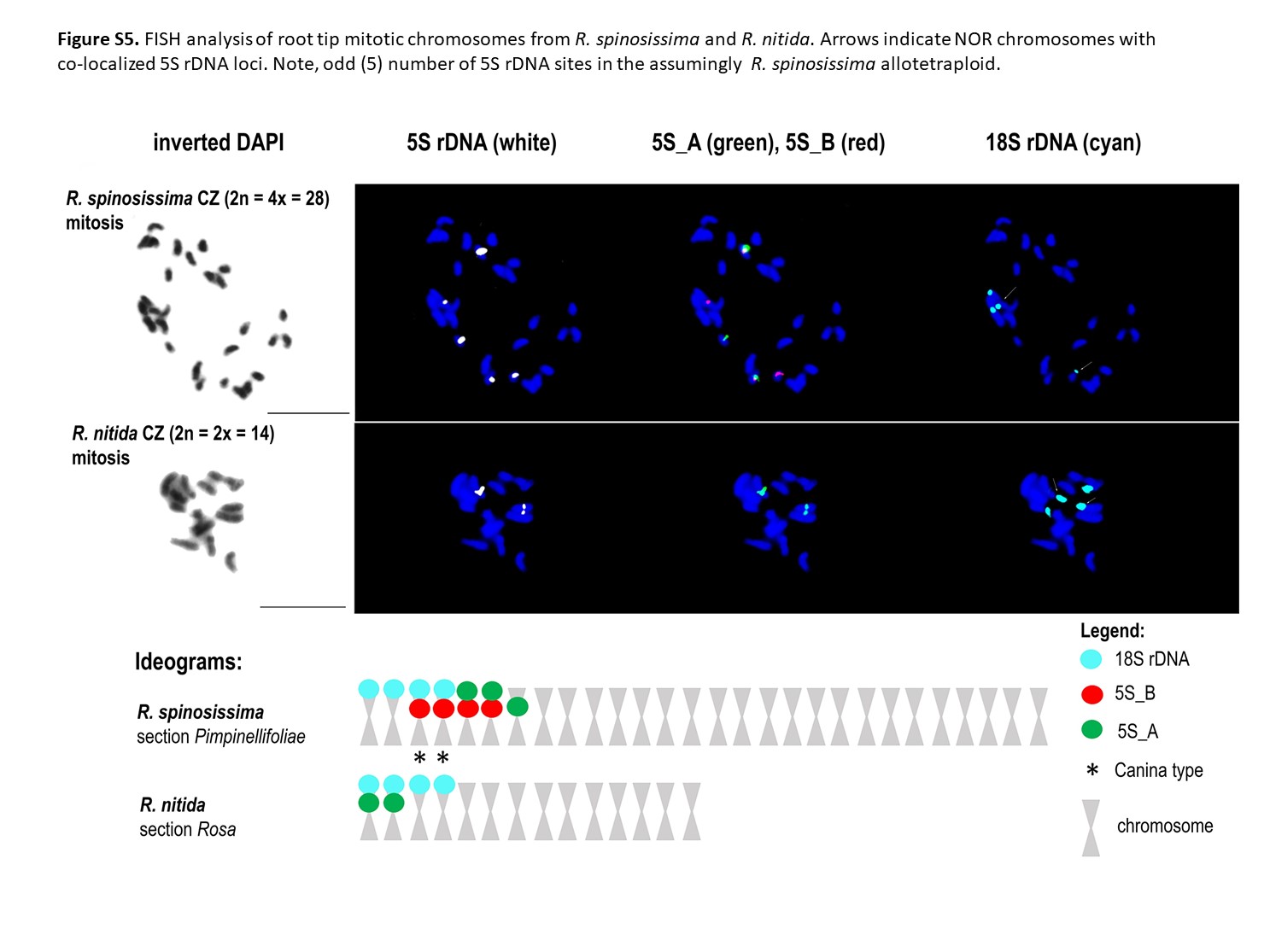

Supplement: Supplementary Figure 5 — FISH analysis of root tip mitotic chromosomes from R. spinosissima and R. nitida. Arrows indicate NOR chromosomes with co-localized 5S rDNA loci. Note odd (5) number of 5S rDNA sites in the assumingly R. spinosissima allotetraploid. [file Image_5.jpg]
